# 2369. kinetics of total and neutralizing SARS-CoV-2 spike antibodies after the 2nd and 3rd dose of the BNT162b2 covid-19 vaccine and association with epidemiological characteristics and breakthrough infection

**DOI:** 10.1093/ofid/ofad500.1990

**Published:** 2023-11-27

**Authors:** Athanasios Michos, Elizabeth-Barbara Tatsi, Filippos Filippatos, Charilaos Dellis, Vasiliki Syriopoulou

**Affiliations:** First Department of Pediatrics, Infectious Diseases and Chemotherapy Research Laboratory, Medical School, National and Kapodistrian University of Athens, ‘Aghia Sophia’ Children’s Hospital, Athens, Greece, Athens, Attiki, Greece; University Research Institute for Maternal and Child Health and Precision Medicine, Athens, Greece, Athens, Attiki, Greece; First Department of Pediatrics, Infectious Diseases and Chemotherapy Research Laboratory, Medical School, National and Kapodistrian University of Athens, ‘Aghia Sophia’ Children’s Hospital, Athens, Greece, Athens, Attiki, Greece; National and Kapodistrian University of Athens, Athens, Attiki, Greece; First Department of Pediatrics, Infectious Diseases and Chemotherapy Research Laboratory, Medical School, National and Kapodistrian University of Athens, ‘Aghia Sophia’ Children’s Hospital, Athens, Greece, Athens, Attiki, Greece

## Abstract

**Background:**

Evidence regarding the kinetics of antibody levels after the 2^nd^ and 3^rd^ doses of the BNT162b2 mRNA COVID-19 vaccine with epidemiological parameters and breakthrough infection is limited.

**Methods:**

We prospectively measured total (TAbs) and neutralizing (NAbs) antibodies against the receptor binding domain of SARS-CoV-2 spike protein in healthcare workers (HCWs) 4 and 8 months after the 2^nd^ and 1 and 8 months after the 3^rd^ dose of the BNT162b2 vaccine. Serum samples from HCWs were tested for TAbs using the Elecsys Anti-SARS-CoV-2 S reagent, while NAbs against wild type SARS-CoV-2 and Omicron variant in non-infected HCWs were measured using the blocking ELISA cPass SARS-CoV-2 neutralization antibody detection kit.

**Results:**

A cohort of 486 vaccinated HCWs with BNT162b2 vaccine with median age (IQR): 49 years (38-56) and 377 (77.6%) females were included in the study. A significant increase in breakthrough infections were observed over time. Specifically, 4 and 8 months after the 2^nd^ dose breakthrough infection happened in 8/486 (1.6%) [median time 3 (IQR:0.75) months] and 11/486 (2.3%) [median time 6 (IQR:6)], respectively. One and 8 months after the 3^rd^ dose breakthrough infection happened in 35/486 (7.2%) [median time 1 (IQR:0) month] and 188/486 (38.7%) [median time 4 (IQR:2) months], respectively. No significant differences in vaccinated HCWs 1 month after the 3^rd^ dose were detected among those who got infected or did not have a breakthrough infection during the next 7 month regarding TAbs [median (IQR): 16611 (13011) vs 17572.5 (14501.0), *p*=0.529] and NAbs [median (IQR): 96.5 (1.7) vs 96.7 (1.9), *p*=0.555], respectively. After infection, HCWs had significantly increased TAbs levels in all time points compared to non-infected vaccinated HCWs **(Table 1**). In non-infected HCWs, a negative association of TAbs and NAbs levels with age was detected after the 2^nd^ and 3^rd^ vaccine dose (*p*< 0.05). No association was found in 8 months after the 3^rd^ dose between antibody levels and demographic and clinical parameters.

**Figure:**
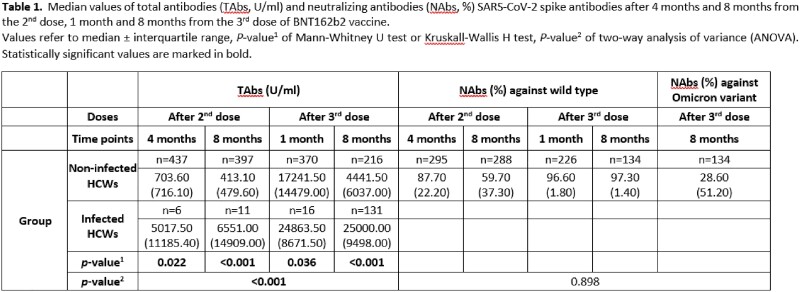

Median values of total antibodies (TAbs, U/ml) and neutralizing antibodies (NAbs, %) SARS-CoV-2 spike antibodies after 4 months and 8 months from the 2nd dose, 1 month and 8 months from the 3rd dose of BNT162b2 vaccine. Values refer to median ± interquartile range, P-value1 of Mann-Whitney U test or Kruskall-Wallis H test, P-value2 of two-way analysis of variance (ANOVA). Statistically significant values are marked in bold.

**Conclusion:**

Findings of the present study indicate that levels of TAbs and NAbs after 3 doses of the BNT162b2 vaccine are not directly associated with the possibility of a breakthrough infection.

**Disclosures:**

**All Authors**: No reported disclosures

